# Bacterial Root Microbiome of Plants Growing in Oil Sands Reclamation Covers

**DOI:** 10.3389/fmicb.2017.00849

**Published:** 2017-05-16

**Authors:** Eduardo K. Mitter, J. Renato de Freitas, James J. Germida

**Affiliations:** ^1^Department of Food and Bioproduct Sciences, University of Saskatchewan, SaskatoonSK, Canada; ^2^Department of Soil Science, University of Saskatchewan, SaskatoonSK, Canada

**Keywords:** oil sands, land reclamation, rhizosphere bacteria, endophytic bacteria, 16S rRNA gene sequencing, NGS analysis

## Abstract

Oil sands mining in northern Alberta impacts a large footprint, but the industry is committed to reclaim all disturbed land to an ecologically healthy state in response to environmental regulations. However, these newly reconstructed landscapes may be limited by several factors that include low soil nutrient levels and reduced microbial activity. Rhizosphere microorganisms colonize plant roots providing hosts with nutrients, stimulating growth, suppressing disease and increasing tolerance to abiotic stress. High-throughput sequencing techniques can be used to provide a detailed characterization of microbial community structure. This study used 16S rRNA amplicon sequencing to characterize the bacterial root microbiome associated with annual barley (*Hordeum vulgare*) and sweet clover (*Melilotus albus*) growing in an oil sands reclamation area. Our results indicate that *Proteobacteria* dominated the endosphere, whereas other phyla such as *Acidobacteria* and *Gemmatimonadetes* were restricted to the rhizosphere, suggesting that plants have the ability to select for certain soil bacterial consortia. The bacterial community in the endosphere compartments were less rich and diverse compared to the rhizosphere. Furthermore, it was apparent that sweet clover plants were more selective, as the community exhibited a lower richness and diversity compared to barley. Members of the family *Rhizobiaceae*, such as *Sinorhizobium* and *Rhizobium* were mainly associated with clover, whereas *Acholeplasma* (wall-less bacteria transmitted by insects) was unique to barley. Genera from the *Enterobacteriaceae* family, such as *Yersinia* and *Lentzea* were also mostly detected in barley, while other genera such *Pseudomonas* and *Pantoea* were able to successfully colonize both plants. Endophytic bacterial profiles varied within the same plant species at different sampling locations; however, these differences were driven by factors other than slope positions or cover management. Our results suggest that bacterial endophytic communities of plants growing in land reclamation systems are a subset of the rhizosphere community and selection is driven by plant factors.

## Introduction

Soil microbial communities represent the greatest known reservoir of biological diversity (Berendsen et al., 2012). However, compared to non-rooted bulk soil, the rhizosphere, which is the narrow zone of soil that is influenced by root exudates, is a ‘hot spot’ for numerous organisms and is considered as one of the most complex ecosystems (Raaijmakers et al., 2009; Bakker et al., 2013; Tkacz et al., 2015). The increased microbial abundance and activities in the rhizosphere environment are due to the release of organic carbon by plant root exudation (Bakker et al., 2013). Soil microorganisms are chemotactically attracted to root exudates, which allow them to proliferate in this carbon rich environment (Raaijmakers et al., 2009). In turn, rhizosphere microbiota can also directly and/or indirectly affect the composition and biomass of plant communities in natural and agricultural ecosystems (Philippot et al., 2013). The complexity of plant–microbe interactions has resulted in a number of studies that revealed profound effects on plant growth, development, nutrition, diseases, and productivity (Mendes et al., 2013). Although the majority of research in plant–microbe interaction focuses on the rhizosphere, microorganisms are also able to readily colonize most plant compartments and plants can also function as filters of soil microorganisms (Chen et al., 2010; Berg et al., 2014).

Microbes residing within plant tissues (the endosphere) for at least part of their lives, whether in leaves, roots or stems, are considered endophytes (Kobayashi and Palumbo, 2000; Turner et al., 2013). Endophytes are thought to be a sub-population of the rhizosphere microbiome and/or that once inside their hosts they change their metabolism and become adapted to their internal environment (Germida et al., 1998; Turner et al., 2013). The best evidence suggests that microbial endophytes enter at lateral root junctions, most likely at naturally occurring cracks, however, they also have characteristics distinct from rhizosphere inhabiting bacteria, suggesting that not all rhizosphere bacteria can enter plants (Turner et al., 2013). Numerous studies suggest that bacterial endophytes can promote host plant establishment and improve plant growth under adverse conditions (Soleimani et al., 2010; Deng et al., 2011; Khan et al., 2011). Bacterial endophytes may also have the ability to control plant pathogens, insects and nematodes, which make them suitable as biocontrol agents (Hallmann and Berg, 2006). In addition, recent studies also suggest that endophytes may play an important role in remediation of contaminated soils and water (Chen et al., 2010; Guo et al., 2010; Xiao et al., 2010; Mastretta et al., 2013).

Plants and microbes have both adapted to use their close association for their mutual benefit (Edwards et al., 2015). Due to the importance of these associations, interactions between microbes and model plants, such as in Rhizobium-legume symbiosis, have been extensively reported in the literature (Child, 1975; Freiberg et al., 1997). However, the diversity of root associated microorganisms in reclamation soils after mining operations are not well understood.

The Athabasca’s oil sands region in northern Alberta are unconventional petroleum deposits where bitumen, a dense and extremely viscous form of petroleum, is found in combination with sand, clay, and water (Yergeau et al., 2012). Covering an area of over 100,000 km^2^, the oil sands yielded 2.3 million barrels of bitumen per day in 2014^1^. These oil deposits, estimated at 169 billion barrels represent the third largest oil reserve in the world and a major resource within Canada’s energy sector (Kannel and Gan, 2012). However, bitumen lies under a total area of 142,000 km^2^ of natural boreal forest, which needs to be removed during mining operations (MacKenzie and Quideau, 2010). Following bitumen extraction, mine tailings are accumulated in settling ponds where tailing sands are precipitated and the water is recycled (Yergeau et al., 2012; Onwuchekwa et al., 2014). Land reclamation strategies in the oil sands are challenging due to the nature of the tailing sands, a generally inappropriate plant growth medium with low nutrient content, high salinity, high pH, low or no organic matter and residual hydrocarbon products (Lefrançois et al., 2010; Naeth et al., 2011). Hence, land reclamation strategies in the oil sands have focused on covering the tailing sands with suitable reclamation material to improve vegetation establishment. A common practice has been the use of peat-mineral soil mix (PMM) to create a suitable plant growth medium and to provide a source of native plants that can facilitate the vegetation natural recovery (Shaughnessy, 2010). Planting of seedlings of the dominant boreal forest tree species and the colonization by pioneer species is essential to improve reclamation strategies and allow the re-establishment of a natural forest (Renault et al., 2004; Lefrançois et al., 2010). In addition, annual barley (*Hordeum vulgare*) is often planted in reclamation landscapes to provide a quick vegetation cover and erosion control (Audet et al., 2015).

Previous studies on oil sands reclamation sites have focused on the shifts on soil microbial community structure and nutrient profiles (MacKenzie and Quideau, 2010) and the impact of nitrogen fixing *Frankia*-inoculated alders on soil quality and dominant root associated microbial communities (Lefrançois et al., 2010). However, given the challenges of land reclamation in the Alberta’s oil sands, and the importance of root associated microbiota for a successful vegetation cover, an in-depth characterization of these microbial profiles is essential to improve current reclamation strategies.

In this study, we used high-throughput 16S rRNA amplicon sequencing to characterize bacterial communities associated with two plant species growing on an oil sands reclamation area. In addition, we have studied the influences of host plants and landforms on the bacterial community composition and structure. Specifically, we aimed to determine whether soil or plant specific factors were the main source influencing bacterial colonization in these plants.

## Materials and Methods

### Sample Collection and Processing

Annual barley (*Hordeum vulgare*), as a planted species, and white sweet clover (*Melilotus albus*), as an unplanted native species, were collected at an oil sands reclamation area of approximately 2.2 km^2^ near Fort McMurray, AB, Canada. Three biological replicates of each plant and attached rhizosphere soil (0–20 cm depth) were collected at different slope positions along two transects (20 sampling locations) (Supplementary Figure S1 and Table S1). The first transect consisted of 10 sampling locations (S1–S10) in the standard cover, which is a cover management area consisting of a 50 cm of peat-mineral mixture on the surface of 100 cm of tailing sands. The second transect also consisted of 10 sampling locations (E1–E10) in the engineered cover, an area of 50 cm of a peat-mineral mixture on top of 120 cm of tailing sands separated from the bottom 30 cm of tailing sands by a geo-clay liner (GCL). The main objective of the GCL added by the industry is to retain the moisture on the top of the cover to improve plant growth and to prevent seepage from compounds on the bottom of the tailing sands from reaching the surface of the plant cover. Samples were collected during the summer of 2014, transported at 4°C and stored at -20°C until processing within the next 48 h. Soil samples were analyzed for soil organic (TOC) and total carbon (TC) by the method from Dhillon et al. (2015) using a LECO CR-12 C Analyzer. Soil organic matter (OM), was analyzed using the dry-ash method (McKeague, 1978). Soil pH was measured in a 2:1 soil: water slurry. Soil available ammonium and nitrate were determined colorimetrically (660 and 520 nm, respectively) according to Laverty and Bollo-Kamara (1988). Available phosphorus and potassium were measured using a modified Kelowna extraction (Qian et al., 1994) and available sulfate by a calcium chloride extraction (McKeague, 1978) (**Table 1**).

**Table 1:** Soil chemical proprieties of peat-mineral samples (0–20 cm) collected in the engineered and standard cover at an oil sands reclamation area near Fort McMurray, AB, Canada.

						Available				
Cover	Slope	Texture	OM	TOC	TC	NH_4_^+^	NO_3_^-^	SO_4_^2-^	PO_4_^2-^	K^+^
			(%)			(mg⋅kg^-1^of soil)				
Standard	Crest	Sandy Loam	11.4ˆab	7.7ˆabc	8.2ˆabc	3.15ˆab	6.0ˆa	70.7ˆab	7.1ˆb	87.0ˆa
	Level	Sandy Loam	4.1ˆb	3.2ˆc	3.7ˆc	2.10ˆb	1.3ˆa	181.3ˆa	7.3ˆb	74.5ˆa
	Midslope	Sandy Loam	11.3ˆab	13.0ˆa	14.2ˆa	5.17ˆab	5.3ˆa	43.3ˆb	8.1ˆb	135.7ˆa
	Upslope	Sandy Loam	8.6ˆab	7.6ˆabc	7.9ˆabc	2.57ˆb	6.3ˆa	96.5ˆab	4.8ˆb	88.3ˆa
	Depression	Sandy Loam	5.7ˆab	5.1ˆbc	5.3ˆbc	2.20ˆb	1.6ˆa	33.0ˆb	3.2ˆb	63.0ˆa
Engineered	Crest	Sandy Loam	11.2ˆab	10.5ˆabc	11.2ˆabc	3.30ˆab	12.4ˆa	52.1ˆb	3.3ˆb	76.5ˆa
	Level	Sandy Loam	12.9ˆa	9.7ˆabc	10.2ˆabc	3.60ˆab	4.9ˆa	22.4ˆb	7.9ˆb	99.7ˆa
	Midslope	Sandy Loam	7.2ˆab	6.3ˆabc	5.7ˆbc	2.75ˆab	4.2ˆa	20.2ˆb	4.8ˆb	57.0ˆa
	Upslope	Sandy Loam	15.7ˆa	6.8ˆabc	6.9ˆabc	5.80ˆa	12.2ˆa	21.0ˆb	5.5ˆb	62.5ˆa
	Lower slope	Sandy Loam	10.8ˆab	13.3ˆab	14.1ˆab	3.45ˆab	8.3ˆa	23.4ˆb	23.6ˆa	94.0ˆa

*Different letters indicate significant differences (LSD *p* ≤ 0.05). OM, organic matter; TOC, total organic carbon; TC, total carbon.*

### Survey of Rhizosphere and Endophytic Bacteria Community

Plant roots and adhering soil (5 g) were placed into an Erlenmeyer flask containing 195 mL of phosphate-buffered saline (PBS) (1.2 g of Na_2_HPO_4_⋅L^-1^, 0.18 g of NaH_2_PO_4_⋅L^-1^, 8.5 g of NaCl⋅L^-1^) buffer and shaken on a rotary shaker (150 rpm) at 22°C for 25 min. After shaking, the remaining slurry was transferred to a 50 mL Falcon centrifuge tube and centrifuged at 2,000 × *g* for 5 min. The supernatant containing PBS buffer was discarded and the rhizosphere soil stored at -80°C for DNA extraction (Dunfield and Germida, 2003). Root material was recovered and transferred into an Erlenmeyer flask containing 100 mL NaClO (1.05% v⋅v^-1^) in PBS and placed on a rotary shaker (150 rpm) at 22°C for 15 min. To remove the remaining NaClO solution, roots were rinsed 10 times with sterile water and 0.1 mL of the final wash was spread on Trypticase soy agar (TSA) plates to check for contamination (Siciliano and Germida, 1999). In addition, a PCR was conducted on the final wash using the 520F/799R2 bacterial primers to ensure root sterilization. Sterile roots were chopped aseptically and stored in sterile tubes at -80°C for DNA extraction. Root nodules from sweet clover plants were removed prior to DNA extraction.

### DNA Extraction

Total endophytic community DNA was extracted from surface disinfected root samples using the PowerPlant^®^Pro DNA Isolation Kit (MoBio Laboratories Inc., Carlsbad, CA, USA) and the rhizosphere soil community DNA was extracted using the MoBio PowerSoil^®^ extraction kit (MoBio Laboratories Inc., Carlsbad, CA, USA). DNA exactions were conducted following the manufacture’s protocols. The DNA yield was quantified using Qubit^®^Fluorometric Quantitation (Invitrogen) and in a SYBR Safe (Invitrogen) 1% agarose gel by comparison with a high DNA mass ladder (Invitrogen) using a Bio-Rad Gel Doc XR System (Bio-Rad Laboratories, Mississauga, ON, Canada).

### High-Throughput 16S rRNA Amplicon Sequencing

To determine the diversity and bacterial community composition in the endosphere and the rhizosphere, DNA samples were submitted for high-throughput sequencing at McGill University and Génome Québec Innovation Centre using Illumina technology. The primer set and PCR protocol used were as described in Edwards et al. (2007). Briefly, PCR amplifications were conducted using the 520F (5′-AGCAGCCGCGGTAAT-3′)/799R2 (5′-CAGGGTATCTAATCCTGTT-3′) primer set that amplifies the V4 region of the 16S rRNA gene. Amplicons with attached Illumina flow cell adapter sequences were added in Illumina MiSeq 2.0 platform in equimolar concentrations. Sample libraries were prepared according to the MiSeq reagent kit preparation guide (Illumina, San Diego, CA, USA), and the sequencing protocol from Caporaso et al. (2010b).

### Bioinformatics and Statistical Analysis

Sequence reads were analyzed using Mothur v. 1.36.0 (Kozich et al., 2013) and the MiSeq standard operating procedure developed by the same laboratory. This analysis process involves the formation of contigs, removal of error sequences and chimera removal. High-quality reads were down-sampled to the smallest sample size and classified with naïve Bayesian classifier implemented in MOTHUR (classify.seqs) using SILVA taxonomy provided by the authors. Sequences from chloroplasts, archaea, eukaryotic organisms were also removed before taxonomic classification. All operational taxonomic units (OTUs) were clustered at a cutoff of 0.03 (97 % similarity). Rarefaction curves values and Simpson diversity were also generated using Mothur software. Chao1 richness, Shannon diversity, and principal coordinate analysis (PCoA) were performed using QIIME (Quantitative Insights Into Microbial Ecology) 1.9.1 (Caporaso et al., 2010a). Heatmap and ternary plots were conducted using by R v.2.15.2 (R Foundation for Statistical Computing^2^) using the VEGAN package (version 2.0–7) and ggtern (version 2.1.4), respectively. Analysis of variance followed by Tukey *post hoc* test and Spearman’s rank correlations were performed using SAS v 9.3 (SAS Institute Inc., Cary, NC, USA).

### Data Deposition

Metagenomic datasets were deposited in the NCBI sequence read archive (SRA) under the submission ID SUB2526072. The metagenomic project can also be accessed in NCBI under GenomeProject ID 381225 (accession PRJNA381225^3^).

## Results

After quality filtering, the Illumina analysis of the V4 region of the 16S-rRNA genes resulted in the recovery of 5,013,100 sequences and 13,107 unique OTUs (3% dissimilarity) across 120 endophytic and 120 rhizosphere bacterial community samples.

**FIGURE 1:**
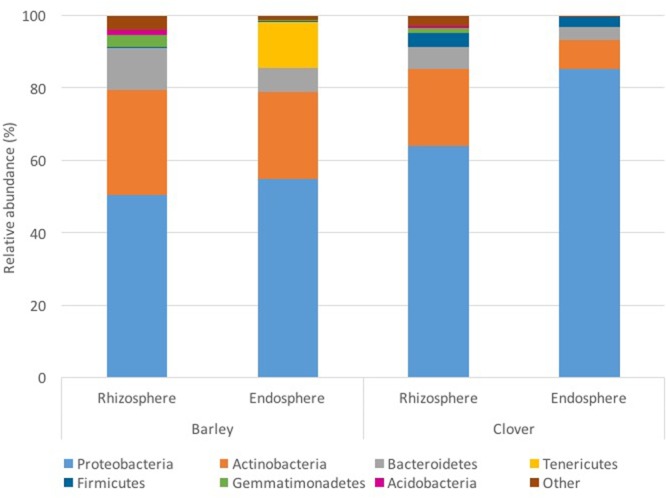
**Analysis of root associated bacterial communities (endosphere and the rhizosphere compartments) at phylum level in barley and sweet clover growing in oil sands reclamation areas**.

The bacterial community consisted of 19 different phyla; however, for most bacterial communities analyzed, only 4 different phyla represented at least 80% of the profile. *Proteobacteria* and *Actinobacteria* were the most abundant phyla observed in all of the samples analyzed (**Figure 1**). In barley plants, *Proteobacteria* represented on average 56% of the endosphere and 49% of the rhizosphere community, whereas they represented 84 and 69% for clover plants, respectively. Although *Proteobacteria* was more abundant in the endosphere, the *Actinobacteria* relative abundance was 24% higher in the rhizosphere for barley and 1.6-fold higher for clover. At phylum level, the two plants analyzed harbored different bacterial communities. Whereas endophytic profiles for barley plants indicated high abundance of *Tenericutes*, sweet clover plants harbored a low abundance of this phylum. *Tenericutes* corresponded to 12% of barley endosphere profiles whereas less than 1% in the rhizosphere and in both rhizo-compartments (endosphere and rhizosphere) of sweet clover plants. However, sweet clover associated bacterial communities also indicated a higher abundance of *Firmicutes* when compared with barley. Overall the rhizosphere communities showed similar profiles between the two plant species analyzed. In addition, although soil physical and chemical proprieties indicated differences between sampling locations (**Table 1**), no significant strong correlations were observed between the most abundant bacterial endophyte phyla and soil chemical parameters (Supplementary Table S2). However, in rhizosphere communities, the phyla *Actinobacteria* indicated significant positive correlations with OM (*R*^2^ = 0.434, *p* ≤ 0.01) total organic carbon (*R*^2^ = 0.370, *p* ≤ 0.05), TC (*R*^2^ = 0.348, *p* ≤ 0.05), available ammonium (*R*^2^ = 0.347, *p* ≤ 0.05), and nitrate (R^2^ = 0.351, *p* ≤ 0.05) (Supplementary Table S3). The phylum *Nitrospira*, although not highly abundant in the rhizosphere compared to *Actinobacteria*, indicated similar positive correlations with soil parameters. *Nitrospira* indicated positive correlations with OM (*R*^2^ = 0.595, *p* ≤ 0.01), total organic carbon (*R*^2^ = 0.381, *p* ≤ 0.05), TC (*R*^2^ = 0.382, *p* ≤ 0.05), available ammonium (*R*^2^ = 0.480, *p* ≤ 0.05), and nitrate (*R*^2^ = 0.452, *p* ≤ 0.05).

**FIGURE 2:**
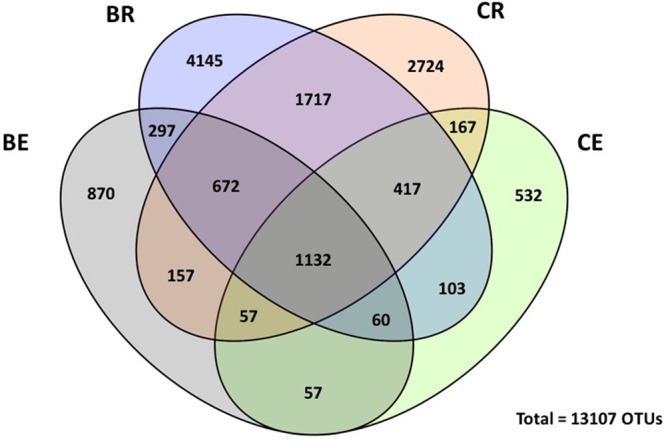
**Venn diagram for endosphere (BE) and rhizosphere (BR) bacterial communities associated with barley and endosphere (CE) and rhizosphere (CR) bacterial communities associated with sweet clover**. Numbers indicated shared unique operational taxonomic units (OTUs) at 0.03 dissimilarity distances after removing singletons involved.

Venn diagram revealed that 1,132 OTUs (which represents 8% of the total number of OTUs) were common to all endophytic and rhizosphere bacterial communities (**Figure 2**). However, 1,717 OTUs (13% of total number of OTUs) were shared only in the different rhizosphere communities and only 57 OTUs (0.4% of total) were shared between endophytic communities. The number of shared OTUs among the two endophytic communities was also the lowest number of OTUs shared between communities. In addition, a total of 8,586 OTUs were unique for rhizosphere samples whereas 1,459 OTUs were unique to the endosphere. As expected, the rhizosphere harbored most of the unique OTUs, in which 4,145 and 2,724 where associated only with barley and sweet clover plants, respectively. Within endophytic communities, 532 OTUs were unique for clover plants and 870 for barley.

**FIGURE 3:**
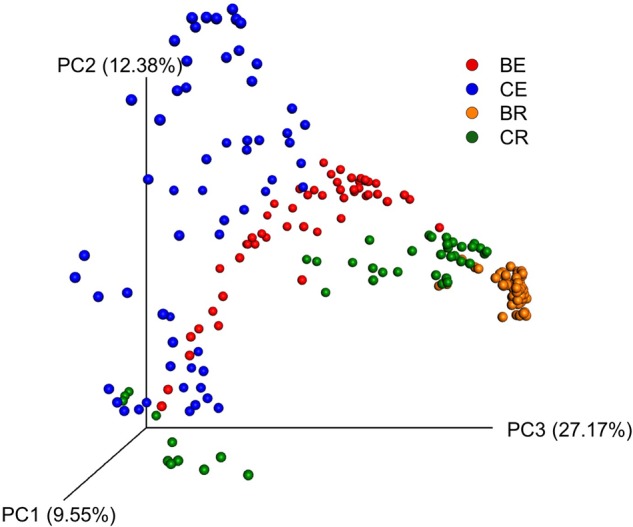
**Principal coordinate analysis (PCoA) based on Bray–Curtis dissimilarity between samples for barley endosphere (BE), clover endosphere (CE), barley rhizosphere (BR), and clover rhizosphere (CR)**.

Similar to Venn diagram, PCoA also indicated differences between bacterial communities (**Figure 3**). PCoA resulted in a 3-dimensional solution in which, PC1 accounted for 9.55% of the variation and PC2 and PC3 for 12.38 and 27.17%, respectively. Based on the different communities, rhizosphere samples were clustered in two regions, one which corresponded to sweet clover associated rhizosphere soil only and another with both barley and sweet clover rhizosphere soil. Overall, results indicate a clear division between the endosphere and rhizosphere compartments. Endophytic communities, however, were more variable between sampling locations when compared to the rhizosphere. In addition, sweet clover endosphere compartments indicated a higher variation among samples when compared to barley. Although clustering was observed based on plant species, no clustering was observed based on cover type and slope positions (Supplementary Figure S2).

**FIGURE 4:**
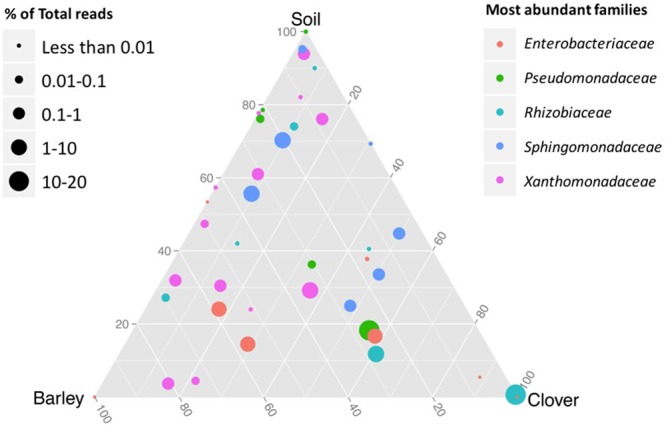
**Ternary plot representing the relative occurrence of individual genus (circles) that are members of the five most abundant families in root samples of sweet clover and barley compared with rhizosphere soil**. Genera enriched in different compartments are colored by taxonomy of the most abundant families. The size of the circles is proportional to the mean abundance in the community.

**FIGURE 5:**
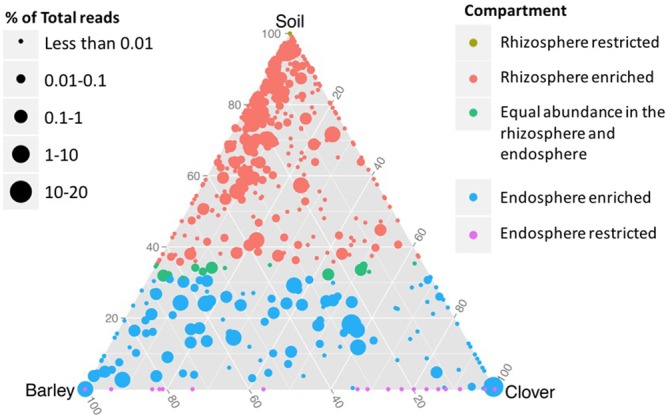
**Ternary plot representing the relative occurrence of individual genus (circles) in root samples of sweet clover and barley compared with rhizosphere soil.** Genera enriched in different compartments are colored according to habitat in which each genus is most frequently associated based on at least 10% enrichment or depletion of soil microbiota in the endosphere. The size of the circles is proportional to the mean abundance in the community.

Based on shared OTUs between different communities (**Figure 2**) and on PCoA (**Figure 3**), our results indicate that although there are differences in the rhizosphere compartments among the two plants analyzed, most of the differences were within the endosphere. Therefore, we analyzed our data using ternary plots based on three main environments: (i) the soil rhizosphere microbiota of both plants, (ii) the endosphere compartment of sweet clover, and (iii) the endosphere of barley (**Figures 4, 5**). According to the most abundant families (**Figure 4**), barley plants harbored a high abundance of *Xanthomonadaceae*, whereas clover plants had a high abundance of *Rhizobiaceae*. Genera from the family *Enterobacteriaceae* were mostly associated with the endophytic communities, whereas *Pseudomonadaceae, Sphingomonadaceae* were associated with both the rhizosphere and endosphere bacterial communities. A ternary plot was used to asses in which compartment each genus is most abundant or restricted (**Figure 5**). Here we categorized each genus based on whether there was a 10% increase or decrease on its relative abundance in endosphere compared with rhizosphere. Our results suggest that only a few genera were restricted by rhizo-compartment, since most of genera can be found in both compartments. Interestingly, barley was more effective at recruiting bacterial genera to its rhizosphere and endosphere than clover.

Chao richness and Shannon diversity indices indicated significant differences between rhizo-compartments (**Table 2**). Both indexes show a lower diversity and richness in the endophytic communities. Additionally, barley rhizosphere and endosphere microbiota had higher richness and diversity when compared to sweet clover.

**Table 2:** Alpha richness and diversity of endophytic and rhizosphere communities associated with barley and sweet clover plants.

		Chao	Shannon
Rhizosphere	Barley (BR)	714.9^a^	4.90^a^
	Clover (CR)	611.6^b^	3.73^b^
Endosphere	Barley (BE)	244.0^c^	3.00^c^
	Clover (CE)	136.4^d^	1.91^d^

*Different letters indicate significant differences (Tukey HSD *p* ≤ 0.05).*

**FIGURE 6:**
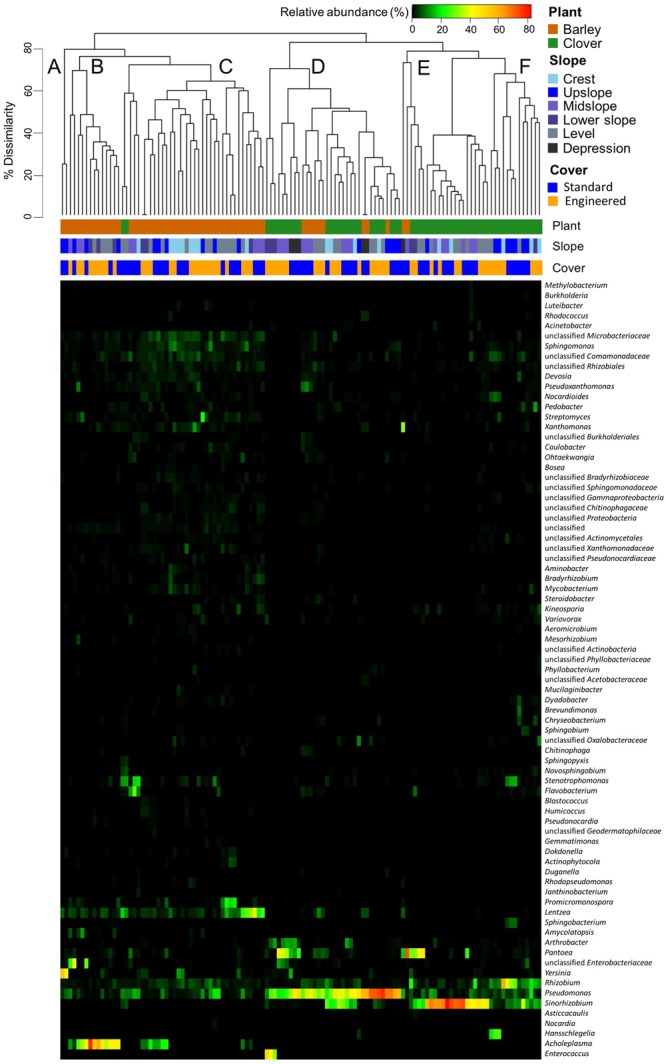
**Heatmap based on relative abundance of sweet clover and barley associated endophytic communities.** Vertical columns represent samples; horizontal rows represent genera that are 2% most abundant in at least one sample. Clustering of samples (top) is based on genera co-occurrence by Bray–Curtis dissimilarity. Letters **(A–F)** indicate different clusters at a 70% dissimilarity cut off.

To investigate the main genera driving differences in the endophytic communities, a heat map using hierarchical cluster based on Bray–Curtis distance was generated using the 2% most abundant genera (**Figure 6**). Our results suggest that bacterial profiles mainly clustered by plant species and not by cover type or by different slope positions.

Six main clusters were observed after a 70% dissimilarity cut off between endophytic community profiles. Based on the cluster profiles, indicator species analysis was conducted to confirm the main genera influencing differences in the bacterial community. The first cluster (A) (left to right) consisted on barley endophytic communities from one sampling location at the upslope in the standard cover. This was the smallest cluster detected and on average the endophytic profiles consisted mainly on *Yersinia* (57%), *Pseudomonas* (7.5%), *Lentzea* (5.6%), *Rhizobium* (2.28%), and *Sphingomonas* (1.49%). The main indicator genus of this cluster was *Yersinia*, which in some samples corresponded to 60% of the profile. Cluster B was limited to barley endophytic communities only, in this cluster the most abundant genera were *Acholeplasma* (46.8 %), unclassified genus from the family *Enterobacteriaceae* (8.5%), *Lentzea* (4.3%), *Pseudomonas* (3.1%), and *Amycolatopsis* (2.7%). *Acholeplasma* was the indicator genus for this cluster, which in some samples this genus could represent up to 80% of the profile. Cluster C corresponded mainly to *Lentzea* (10.4%), *Pseudomonas* (6.4%), unclassified genus from the family *Microbacteriaceae* (4.3%), *Rhizobium* (4.3%), and *Acholeplasma* (3.88%). In this cluster *Lentzea* was the indicator genus for this cluster which it could represent up to 50% of the bacterial profile in some samples. In addition, cluster C was one of the clusters that represented most of the endophytic profiles for barley plants. With the exception of a few samples, no particular genus corresponded to more than 50% of the community structure in this cluster. Cluster D consisted of 76% of sweet clover plants and 24% of barley. On average the most abundant genera in this cluster were *Pseudomonas* (50.1 %), *Sinorhizobium* (7.8%), *Pantoea* (7.5%), *Enterococcus* (4.3%), and *Arthrobacter* (3.8%). *Pseudomonas* was also detected as the indicator genus of cluster D. Cluster E was detected as a small cluster limited to sweet clover plants with *Pantoea* as the indicator genus. On average, this cluster mainly consisted by *Pantoea* (45.7%), *Sinorhizobium* (13.4%), *Pseudomonas* (6.87%), *Xanthomonas* (6.71%), and *Rhizobium* (6.61 %). Cluster F also was limited to sweet clover plants, however, with a dominant *Sinorhizobium* endophytic community. *Sinorhizobium* was the main indicator genus of this cluster and it represented up 90% of the bacterial profile in some samples. The most abundant genera of this cluster were *Sinorhizobium* (44.2%), *Rhizobium* (12.9%), *Pseudomonas* (6.2%), *Hansschlegelia* (2.9%), and unclassified genus from the family *Comamonadaceae* (2.1%).

To investigate rhizosphere associated bacterial communities, a heat map was also generated using hierarchical cluster based on Bray–Curtis distance (Supplementary Figure S3). Rhizosphere bacterial profiles also clustered mainly on different plant species. As expected, clustering dissimilarities were much lower when compared to endophytic communities. Three main clusters were observed after a 40% dissimilarity cut off. Cluster A was limited to sweet clover plant species only, the most dominant genus in this profile is *Pseudomonas* (58%), followed by *Arthrobacter* (12.3%) and *Pantoea* (9.1%). Cluster B also was limited to sweet clover species; however, this cluster was mainly detected by an equal abundance of unclassified genus from the order *Bacillales* (17%), *Pantoea* (15%), and *Acinetobacter* (14%). However, most of rhizosphere profiles were represented in cluster C and interestingly all barley profiles were on this cluster. In cluster C, the most abundant genera detected were *Arthrobacter* (6.5%), *Sphingomonas* (5.3%), and an unclassified genus from the order *Rhizobiales* (4.6%). Also, in this cluster none of the genera identified represented more than 40% of the profile and no dominant genus could be observed. This cluster also included a group of sweet clover associated rhizosphere bacterial profiles mainly differentiated from other samples within the cluster by the abundance of *Stenotrophomonas* (9.1%).

## Discussion

The data presented here provides new insights on plant microbe interactions in reclamation sites, as most previous studies have focused on the microbial communities in tailing ponds (Yergeau et al., 2012) and on the overall soil microbial biomass in oil sands reclamation sites (MacKenzie and Quideau, 2010). Furthermore, very few studies focused on endophytic communities in oil sands reclamation covers using culture-independent methods (Lefrançois et al., 2010). In an attempt to unravel the root associated bacterial microbiome of plants growing in reclamation soils, we used 16S rRNA high-throughput amplicon sequencing to characterize endophytic and rhizosphere bacterial communities associated with two plant species in one of the Athabasca oil sands reclamation sites.

Illumina MiSeq of PCR amplicons and sequence analyses revealed that both endophytic and rhizosphere bacterial profiles varied considerably across the different sampling locations. Our data also suggest that changes in the microbiome are mainly due to different rhizo-compartments (rhizosphere and endosphere) and host plants. Similar results were observed by Ofek-Lalzar et al. (2014), who studied the rhizoplane bacterial communities associated with wheat and cucumber and found that variability was correlated with rhizo-compartment at a higher extent and different host plant at a lesser extent. In our study, we observed a lower diversity in the endosphere compartment compared to the rhizosphere. Hence, as previously reported in Germida et al. (1998) and Edwards et al. (2015), our data also suggests that endophytic root colonization is not a passive process and that both sweet clover and barley plants have the ability to select for certain soil microbial consortia. The enrichment for a subset of selected dominant phyla in the endosphere compartment was also consistent with Shannon diversity and the Chao richness analysis. Siciliano and Germida (1999) also reported changes in the abundance of certain genera in the endosphere when compared to the rhizosphere and a lower diversity in the endosphere.

Differences in bacterial community profiles in our data were detected at a broad taxonomic level such as at the phylum level. Our results indicate that there was an increase in the relative abundance of *Proteobacteria* in the endophytic community of both plants when compared to the rhizosphere. *Proteobacteria* were previously described as effective rhizosphere and root colonizers in several plants such as rice (Edwards et al., 2015), smooth cordgrass (Hong et al., 2015), and wheat (Ai et al., 2015) due to their high ability to utilize root exudates (Fierer et al., 2007). *Proteobacteria* are known to respond rapidly to carbon sources, and are generally considered to be r-strategists and fast-growing bacteria (Peiffer et al., 2013). The enrichment of *Proteobacteria* spp. in root compartments, mostly in sweet clover plants, was previously suggested in the literature in tomato (Yao and Allen, 2006) and in grapevine (Zarraonaindia et al., 2015) as a response to chemotaxis via photoassimilates secreted by root cells (Bulgarelli et al., 2013).

Similar to other studies using *Arabidopsis thaliana* (Lundberg et al., 2012) and rice plants (Edwards et al., 2015), our results revealed that several phyla common in the rhizosphere (*Acidobacteria, Verrucomicrobia*, and *Gemmatimonadetes*), were almost absent in the endosphere. In fact, our results indicate that relative abundance of *Acidobacteria* is below 1% in endophytic profiles at some sampling locations. However, differently from Hong et al. (2015) and Edwards et al. (2015), we found a high abundance of *Actinobacteria* in all rhizosphere profiles, which is in agreement with Bodenhausen et al. (2013) and Sugiyama et al. (2014). According to Bulgarelli et al. (2013), *Actinobacteria* is considered a specific bacterial taxon that responds favorably to organic carbon substrate addition and the high abundance of this phylum has been observed in both rhizosphere and endosphere compartments of different plant species. Also, differently from the *Arabidopsis thaliana* root microbiome studied in Lundberg et al. (2012), we found that both sweet clover and barley plants harbored a higher relative abundance of *Firmicutes* in rhizosphere profiles when compared with the endosphere. According to Bulgarelli et al. (2013), *Firmicutes* dominate both rhizo-compartments, however, the dominance in the endosphere can only be observed in certain plants. Furthermore, we observed that the phylum *Tenericutes* was only detected in the endosphere compartment of barley plants. *Tenericutes* is a phylum that contains the class Mollicutes, characterized by the absence of a cell wall (Montagna et al., 2015). Recently, Rivera-Tapia et al. (2002) have reported that *Tenericutes* are known to colonize the gut of animals, insects and plants.

Although soil chemical analysis revealed significant differences between sampling locations, we only observed strong correlations between these parameters and bacterial phyla in the rhizosphere. Some of these correlations have been previously suggested in the literature, such as positive correlations between soil organic matter, total organic carbon, and the abundance of *Actinobacteria* (Li et al., 2012; Embarcadero-Jiménez et al., 2015). However, in endophytic profiles, our results suggest that these communities may be driven by factors other than the soil chemical parameters analyzed in our study.

The number of shared unique OTUs in shown by Venn diagram suggests that rhizosphere samples contained the majority of OTUs in the dataset, which confirms that soil serves as a primary reservoir for potential endophytes (Germida et al., 1998; Zarraonaindia et al., 2015). Furthermore, differences in rhizo-compartments were also observed in PCoAs. Here, rhizosphere soil samples differentiated from its respective endophytic bacterial communities, as previously reported in the literature (Lundberg et al., 2012; Zarraonaindia et al., 2015). Additionally, bacterial community profiles analyzed by PCoA indicated clustering regions containing a low variability between samples in rhizosphere profiles and a high variability in endophytic profiles. During land reclamation activities, no soil or seed inoculation was conducted, hence bacterial profiles in all rhizo-compartments studied here corresponded to naturally occurring indigenous communities.

Previous studies (Germida et al., 1998; Ofek-Lalzar et al., 2014) suggested that plant factors play a dominating role in the endophytic community composition and bacterial communities vary between plant species. Several studies have suggested that different root exudates produced by different plant species may affect distinct root associated microbial populations (Phillips et al., 2012). Based on these evidences, ternary plots were generated using the mean relative abundance from each genus in each root endosphere compartments and the rhizosphere soil of both plants combined. Here, although plant factors may actively select for certain soil microbial consortia, our results indicate that sweet clover plants were more restrictive when compared to barley. In addition, sweet clover plants were more closely associated with members of the family *Rhizobiaceae* while barley plants harbored a high abundance of *Xanthomonadaceae*. The economic importance of *Rhizobiaceae* and its potential in nitrogen fixation have been extensively reported in the literature (Ludwig, 1980; Long, 1996) as well as their ability to colonize the root interior of leguminous plants (e.g., alfalfa and sweet clover plants) (Bromfield et al., 2010). Nitrogen fixers associated with naturally occurring plant species can facilitate vegetation development through addition of atmospheric nitrogen to the system and could alleviate potential nutrient limitation in reclamation areas (Lefrançois et al., 2010). In our dataset, the family *Xanthomonadaceae* contains the genus *Xanthomonas*, which some are known as plant pathogens (Soares et al., 2010), but mainly *Stenotrophomonas*, which are capable of great metabolic versatility and are colonizers of soil and plants (Ryan et al., 2009). *Stenotrophomonas* were previously isolated in barley rhizosphere soil (Caesar-TonThat et al., 2007), reported as a multifunctional plant growth-promoting rhizobacterium (PGPR) (Alavi et al., 2013) and to induce antagonistic behavior against soil-borne plant pathogens (Dunne et al., 1998). Our results also suggest a high abundance of the family *Enterobacteriaceae* associated with both endosphere plant compartments. Members of the *Enterobacteriaceae* family are often associated as human pathogens, however, this family consists of a large group distributed in many environments (Yousaf et al., 2011). *Enterobacteriaceae* are widespread in several plant systems and some have been suggested as beneficial plant-associated bacteria that can promote plant growth (Kämpfer et al., 2005) and biocontrol activity (Chernin et al., 1995). Therefore, the data presented in our study strongly suggest that the two plants analyzed supported the enrichment of different bacterial taxa. Alternatively, plant factors such as root exudation, may drive the selection of different bacterial taxa (Phillips et al., 2012).

Since most of our data indicated differences occurring mainly in endosphere compartments, we used heat map analysis for a finer and more specific comparison between profiles. Our results using heat map also support previous analysis in the dataset in which endophytic bacterial profiles differentiated mainly among plant species. Although these profiles varied within the same plant species at each sampling location, differences in these profiles may be driven to factors other than slope positions or cover managements. Within the endophytic community, *Sinorhizobium, Pseudomonas, Rhizobium, Acholeplasma, Lentzea, Pantoea*, and *Yersinia* were the main genera driving these differences. *Sinorhizobium* have been previously reported as nitrogen-fixing bacterial endophytes of alfalfa, beans, and sweet clover (Bromfield et al., 2010; Dudeja et al., 2012). Sweet clover is a fast-growing legume and our results suggest that *Sinorhizobium* corresponds to a significant share of endophytes associated with this plant. *Sinorhizobium* can be free-living in the soil or form nitrogen-fixing nodules on the roots of leguminous plants such as the genera *Melilotus* (Biondi et al., 2009). Sweet clover plants in our study may rely on the association with *Sinorhizobium* spp. to grow in reclamation soils. However, the high dominance of this genus is not always observed in sweet clover endophytic profiles analyzed in our study. *Pseudomonas* species can successfully colonize both barley and clover plants, although most of the profiles with a high relative abundance of *Pseudomonas* were observed in sweet clover plants. These results were expected, as it was previously observed that *Pseudomonas* ssp. are common colonizers of the plant interior (Moore et al., 2006; Ofek-Lalzar et al., 2014). Although this genus contains pathogenic species, a wide range of *Pseudomonas* ssp. are known for PAH degradation (Germaine et al., 2009), potential heavy metal extraction enhancement (Rajkumar et al., 2009), and PGPR (Bhattacharyya and Jha, 2012). *Rhizobium* species were most commonly found in sweet clover plants and with *Sinorhizobium*, whereas *Acholeplasma* species were restricted to barley plants. *Acholeplasma* are wall-less bacteria from the phylum *Firmicutes* and close relatives of *Phytoplasmas*, whereas *Acholeplasma*s are not known to be pathogenic (Kube et al., 2014). *Acholeplasmas* are also known to colonize the guts and hemolymph of insects (Tully et al., 1988) and transmission to plants occurs when these insects feed on plant tissues (Bonnet et al., 1991). In our study, barley plants may be more susceptible to insect feeding than clover plants and, hence, a higher incidence of *Acholeplasma* species in barley. Although most of endophytic profiles analyzed corresponded to clusters mainly driven by *Sinorhizobium, Pseudomonas, Rhizobium*, and *Acholeplasma*, we also observed smaller clusters with a high incidence of *Pantoea, Lentzea*, and *Yersinia*. *Pantoea* is a gram-negative bacteria of the family *Enterobacteriaceae* first identified by Gavini et al. (1989), which can be human and clinical strains, epi- and endophytes or merely present in water and soil samples (Brady et al., 2008). Previous studies have also demonstrated the application of *Pantoea* in heavy metal biosorption (Ozdemir et al., 2004), plant growth promotion (Feng et al., 2006), and phenolic compounds degradation (Dastager et al., 2009). Similar to *Pantoea, Lentzea*, and *Yersinia* are also members of the family *Enterobacteriaceae*. *Lentzea* is a genus of mesophilic actinomycetes first identified by Yassin et al. (1995) and later identified as capable of biodegradation of aliphatic polyester poly(lactide) (Jarerat et al., 2002; Tokiwa and Jarerat, 2004). The genus *Yersinia*, although commonly known as human pathogens (Perry and Fetherston, 1997), consists of 15 species of mostly harmless environmental organisms residing in the plant interior, soil and water (Hallmann and Berg, 2006; Ayyadurai et al., 2010). Lawson and Afenu (2013) have also identified *Yersinia* spp. isolates with potential capabilities of degrading diesel oil. Both *Yersinia* and *Lentze*a were mostly detected in barley endophytic profiles, which may suggest these organisms are adapted to survive as part of their lifecycle in barley, but encounter a less favorable environment in sweet clover.

To the best of our knowledge, this study provided the first in-depth analysis of bacterial endophytic profiles of plants growing in oil sands reclamation areas. Consistent with prior findings based on high-throughput amplicon sequencing (Bulgarelli et al., 2012; Ofek-Lalzar et al., 2014), our results confirm that rhizo-compartments produce the strongest differentiation of root associated bacterial communities. In addition, host plants also account as main driving factors affecting the endophytic microbiome. A lower diversity in the endosphere compartment and the depletion or enrichment of certain bacteria strongly suggests that plant factors select for certain soil bacterial consortia. Endophytic profiles studied here also revealed that sweet clover plants were more selective than barley. Whereas members of the family *Rhizobiaceae*, such as *Sinorhizobium* and *Rhizobium* were mainly associated with clover, *Acholeplasma* was unique to barley. *Yersinia* and *Lentzea* were also mostly detected in barley, although *Pseudomonas* and *Pantoea* were able to successfully colonize both plants. Endophytic bacterial profiles also varied within the same plant species at different sampling locations; however, these differences were driven by factors other than the soil parameters analyzed in our study. Future studies will be focused on determining the mechanisms driving root associated communities and functional aspects within this microbiome to improve plant growth in reclamation areas.

## Author Contributions

EM contributed for experimental design, sampling, lab analysis, interpretation and analysis of data and writing the manuscript. JdF contributed in experimental design, interpretation of data and manuscript revisions. JG contributed in experimental designs, interpretation of data and manuscript revisions.

## Conflict of Interest Statement

The authors declare that the research was conducted in the absence of any commercial or financial relationships that could be construed as a potential conflict of interest.

## Footnotes

1 http://www.energy.alberta.ca/Oilsands/791.asp

2 http://www.R-project.org

3 http://www.ncbi.nlm.nih.gov/bioproject/381225
