# Supplementary material for: Bacterial Root Microbiome of Plants Growing in Oil Sands Reclamation Covers
Source: Front Microbiol. 2017 May 16;8:849. doi: 10.3389/fmicb.2017.00849 (PMC5432656; doi:10.3389/fmicb.2017.00849)
Supplement: Supplementary file 1 [file Data_Sheet_1.docx]

Supplementary Material

Bacterial root microbiome of plants growing in oil sands reclamation covers

Eduardo K. Mitter, J. Renato de Freitas, James J. Germida.

*** Correspondence:** Eduardo K. Mitter: ekm412@mail.usask.ca


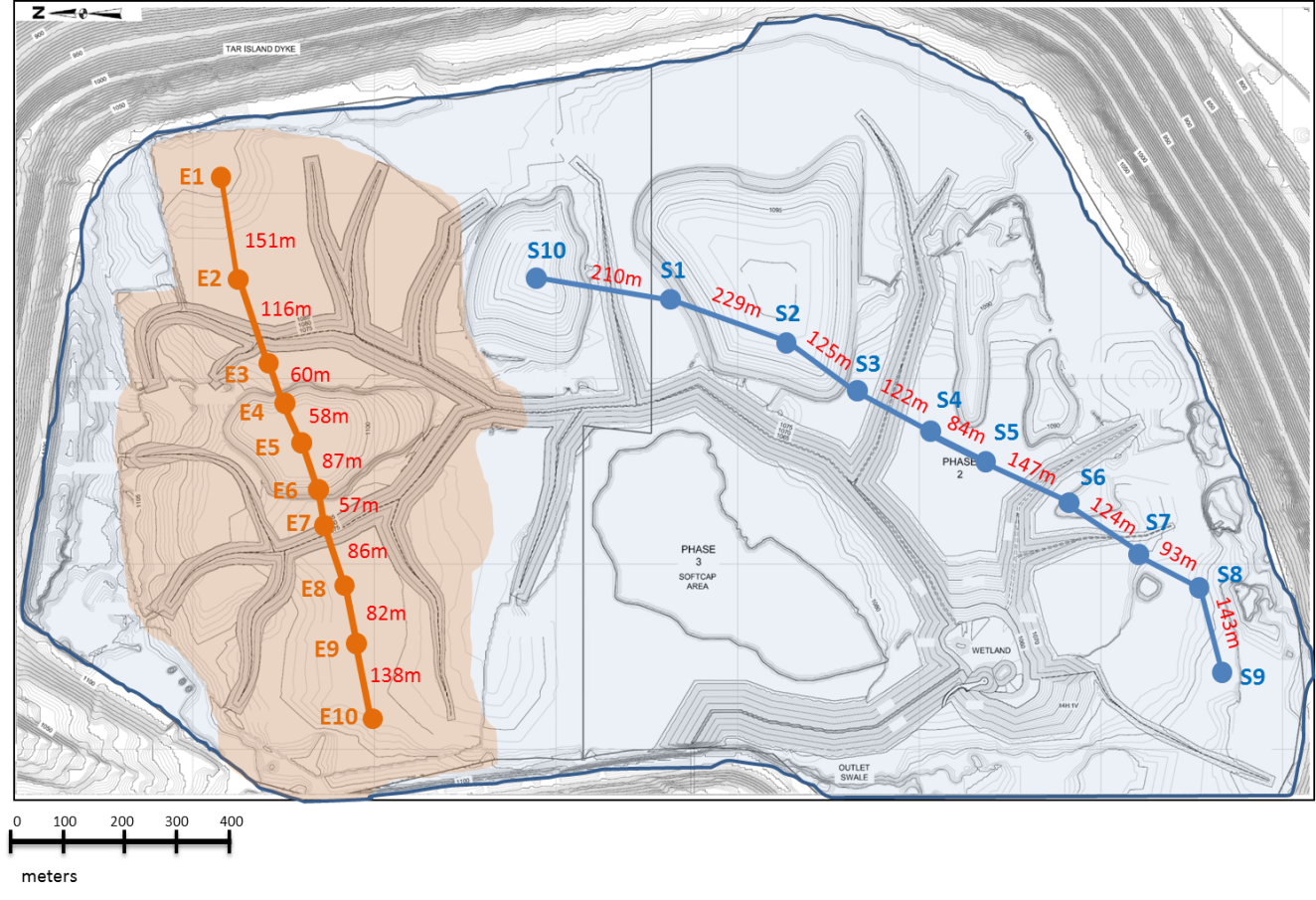


**Fig. S1.** Transects of sampling locations along the engineered (E1-E10) and standard cover (S1-S10) in an oil sands reclamation area near Fort McMurray, Alberta, Canada.

**Table S1.** Landforms and elevation of sampling locations.

| **Location** | **Cover** | **Elevation (m)** | **Landform** |
| --- | --- | --- | --- |
| S1 | Standard | 307.89 | Midslope |
| S2 | Standard | 308.25 | Midslope |
| S3 | Standard | 304.14 | Depression |
| S4 | Standard | 304.00 | Upslope |
| S5 | Standard | 305.72 | Crest |
| S6 | Standard | 307.21 | Level |
| S7 | Standard | 304.02 | Upslope |
| S8 | Standard | 304.05 | Midslope |
| S9 | Standard | 308.45 | Level |
| S10 | Standard | 312.23 | Upslope |
| E1 | Engineered | 310.04 | Level |
| E2 | Engineered | 305.23 | Crest |
| E3 | Engineered | 306.00 | Lower slope |
| E4 | Engineered | 310.94 | Upslope |
| E5 | Engineered | 315.26 | Crest |
| E6 | Engineered | 310.04 | Midslope |
| E7 | Engineered | 306.43 | Lower slope |
| E8 | Engineered | 307.16 | Level |
| E9 | Engineered | 305.54 | Level |
| E10 | Engineered | 306.17 | Level |

|  | pH | OM | TOC | TC | NH_4_^+^ | NO_3_^-^ | SO_4_^2-^ | PO_4_^2-^ | K^+^ | EC |
| --- | --- | --- | --- | --- | --- | --- | --- | --- | --- | --- |
| *Proteobacteria* | 0.093 | 0.044 | -0.024 | -0.015 | 0.151 | 0.105 | 0.112 | -0.216 | 0.091 | 0.052 |
| *Actinobacteria* | 0.013 | 0.031 | -0.147 | -0.162 | -0.140 | 0.028 | -0.085 | 0.007 | -0.158 | 0.132 |
| *Bacteroidetes* | 0.060 | -0.050 | -0.146 | -0.148 | -0.093 | 0.033 | 0.309 | -0.051 | 0.175 | 0.160 |
| *unclassified* | -0.148 | 0.003 | -0.195 | -0.202 | -0.064 | 0.033 | 0.062 | 0.033 | 0.070 | 0.006 |
| *Gemmatimonadetes* | -0.120 | 0.192 | -0.139 | -0.154 | 0.037 | 0.161 | 0.106 | -0.172 | 0.143 | 0.164 |
| *Firmicutes* | 0.142 | 0.009 | -0.195 | -0.202 | 0.044 | 0.143 | -0.066 | -0.215 | -0.244 | 0.167 |
| *Acidobacteria* | 0.060 | 0.107 | -0.059 | -0.066 | 0.014 | 0.287 | 0.119 | -0.114 | **0.350*** | 0.102 |
| *Verrucomicrobia* | 0.019 | -0.134 | -0.268 | -0.276 | -0.266 | -0.011 | 0.097 | 0.069 | 0.292 | 0.138 |
| *Tenericutes* | -0.148 | 0.085 | -0.021 | -0.016 | 0.022 | 0.063 | -0.031 | 0.124 | -0.144 | -0.115 |
| *Armatimonadetes* | -0.064 | 0.045 | -0.009 | 0.005 | 0.017 | 0.106 | 0.069 | 0.023 | 0.167 | 0.076 |
| *Nitrospira* | -0.089 | 0.266 | 0.247 | 0.246 | 0.243 | 0.132 | 0.076 | -0.036 | **0.318*** | -0.186 |
| *Chlamydiae* | -0.094 | -0.055 | 0.115 | 0.137 | -0.006 | 0.110 | 0.145 | 0.109 | 0.116 | -0.045 |
| *Chloroflexi* | 0.007 | 0.118 | -0.078 | -0.091 | 0.063 | 0.081 | 0.097 | -0.193 | 0.002 | -0.055 |
| *Planctomycetes* | 0.004 | -0.049 | -0.017 | -0.017 | -0.055 | -0.170 | 0.087 | -0.126 | -0.076 | -0.002 |
| *TM7* | 0.027 | -0.014 | 0.037 | 0.050 | -0.043 | 0.019 | 0.127 | -0.012 | 0.179 | -0.087 |
| *Spirochaetes* | 0.016 | -0.183 | 0.047 | 0.047 | -0.243 | -0.063 | -0.067 | 0.176 | 0.035 | -0.095 |
| *Deinococcus–Thermus* | -0.008 | 0.087 | -0.003 | -0.003 | **0.340*** | 0.105 | 0.116 | **-0.328*** | -0.057 | -0.180 |
| *Fusobacteria* | -0.209 | -0.125 | -0.208 | -0.236 | -0.209 | -0.153 | 0.042 | 0.236 | 0.097 | -0.266 |

**Table S2**. Correlation indicating Spearman’s r values relating endophytic bacterial phyla and soil parameters. Statistically significant correlations are indicated in bold type, * and ** denote *p* values ≤ 0.05 and ≤ 0.01, respectively. OM = organic matter, TOC = total organic carbon, TC = total carbon, EC = electrical conductivity.

**Table S3.** Correlation indicating Spearman’s r values relating rhizosphere bacterial phyla and soil parameters. Statistically significant correlations are indicated in bold type, * and ** denote p values ≤ 0.05 and ≤ 0.01, respectively. OM = organic matter, TOC = total organic carbon, TC = total carbon, EC = electrical conductivity.

|  | pH | OM | TOC | TC | NH_4_^+^ | NO_3_^-^ | SO_4_^2-^ | PO_4_^2-^ | K^+^ | EC |
| --- | --- | --- | --- | --- | --- | --- | --- | --- | --- | --- |
| *Proteobacteria* | 0.134 | -0.115 | -0.027 | -0.036 | -0.034 | -0.054 | -0.103 | 0.122 | 0.092 | -0.113 |
| *Actinobacteria* | -0.098 | **0.434**** | **0.370*** | **0.348*** | **0.347*** | **0.351*** | -0.237 | -0.155 | 0.072 | -0.124 |
| *Bacteroidetes* | 0.169 | -0.019 | -0.025 | -0.026 | 0.065 | -0.027 | 0.070 | -0.191 | 0.077 | 0.039 |
| *unclassified* | 0.064 | 0.030 | -0.023 | -0.008 | 0.107 | -0.058 | 0.206 | -0.240 | 0.040 | 0.040 |
| *Gemmatimonadetes* | 0.067 | 0.039 | -0.043 | -0.020 | 0.026 | 0.108 | 0.177 | -0.183 | -0.126 | 0.206 |
| *Firmicutes* | 0.073 | 0.023 | -0.108 | -0.113 | 0.163 | -0.050 | -0.017 | -0.044 | -0.031 | -0.195 |
| *Acidobacteria* | -0.068 | 0.156 | 0.167 | 0.184 | 0.158 | 0.002 | 0.060 | 0.021 | 0.137 | -0.154 |
| *Verrucomicrobia* | 0.086 | 0.090 | 0.006 | 0.025 | 0.269 | 0.034 | 0.173 | **-0.317*** | 0.002 | 0.014 |
| *Tenericutes* | 0.254 | -0.259 | -0.209 | -0.202 | -0.050 | -0.212 | 0.194 | -0.097 | -0.096 | 0.082 |
| *Armatimonadetes* | 0.158 | -0.063 | 0.041 | 0.062 | 0.068 | -0.035 | 0.220 | -0.106 | 0.184 | -0.087 |
| *Nitrospira* | -0.323 | **0.595**** | **0.381*** | **0.382*** | **0.480**** | **0.452*** | -0.249 | -0.134 | -0.009 | -0.275 |
| *Chlamydiae* | 0.045 | 0.040 | 0.140 | 0.129 | 0.065 | 0.064 | -0.013 | -0.082 | 0.059 | 0.015 |
| *Chloroflexi* | -0.022 | -0.070 | -0.101 | -0.097 | -0.104 | -0.126 | 0.289 | -0.097 | 0.036 | 0.102 |
| *Planctomycetes* | -0.142 | 0.119 | 0.021 | 0.018 | -0.036 | 0.057 | 0.045 | -0.003 | 0.055 | 0.026 |
| *Chlorobi* | 0.006 | **0.325*** | 0.205 | 0.205 | 0.308 | **0.344**** | -0.220 | -0.220 | -0.145 | 0.153 |
| *TM7* | -0.034 | 0.063 | 0.101 | 0.102 | 0.055 | 0.121 | 0.210 | 0.276 | 0.238 | -0.290 |
| *Spirochaetes* | 0.028 | -0.199 | -0.074 | -0.093 | 0.007 | -0.178 | 0.162 | -0.019 | 0.180 | -0.278 |

**
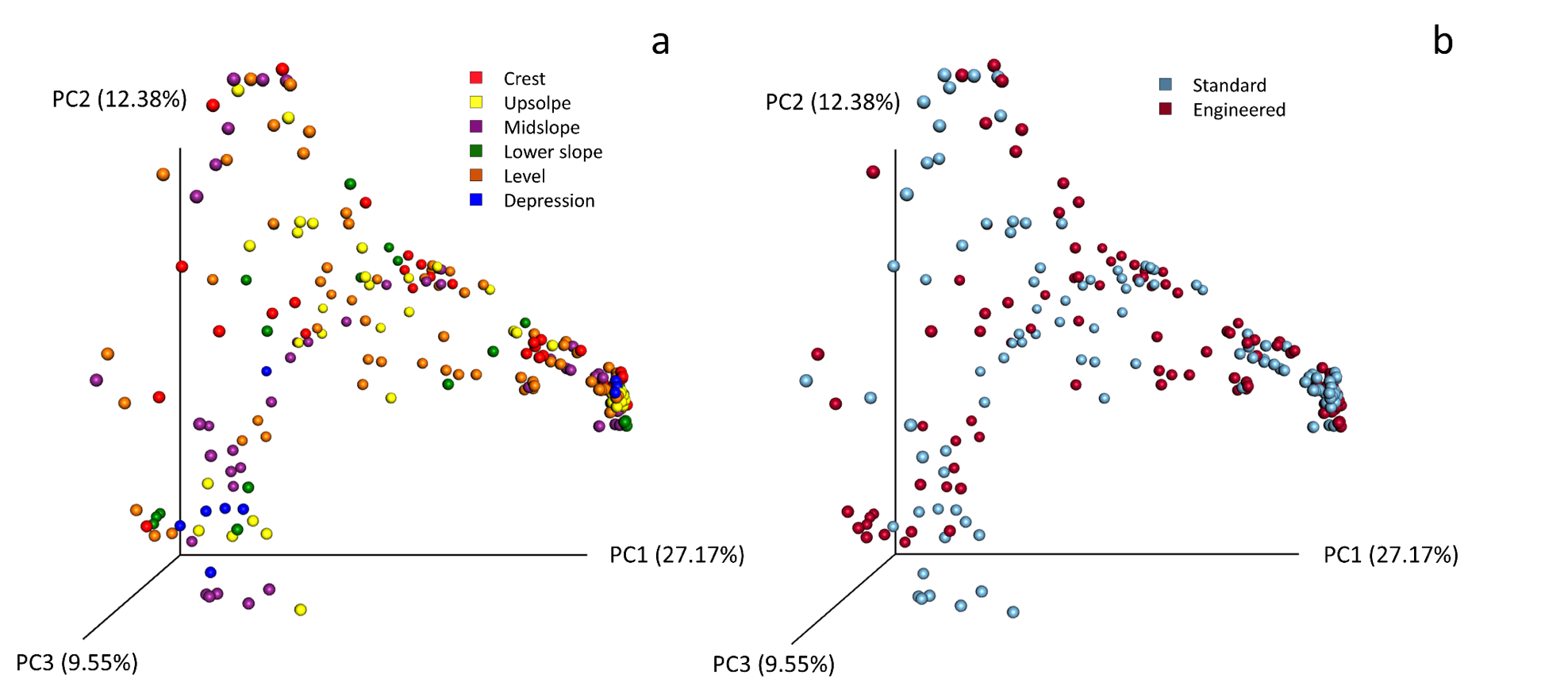
**

**Fig. S2.** Principal Coordinate Analysis (PCoA) based on Bray-Curtis dissimilarity between samples based on different slope communities (a) and cover managements (b)**.**


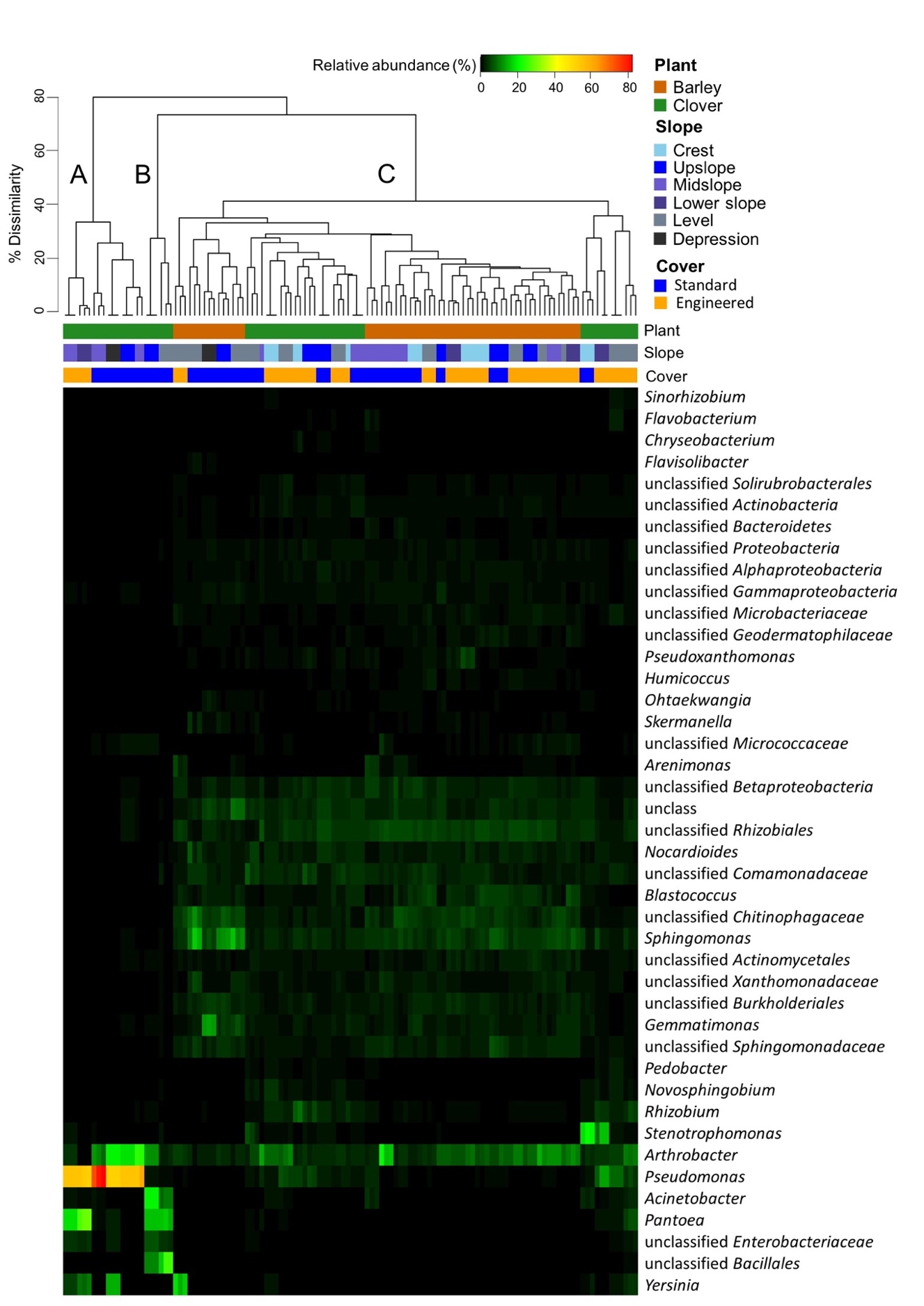


**Fig. S3.** Heatmap based on relative abundance of sweet clover and barley associated rhizosphere communities. Vertical columns represent samples; horizontal rows represent genera that are 2% most abundant in at least one sample. Clustering of samples (top) is based on genera co-occurrence by Bray-Curtis dissimilarity. Letters (A-C) indicate different clusters at a 40% dissimilarity cut off.
